# Synthesis and Photochromic Properties of Diarylethene Derivatives with Aggregation-Induced Emission (AIE) Behavior

**DOI:** 10.3390/ma18112520

**Published:** 2025-05-27

**Authors:** Jiaxin Guo, Haoyuan Yu, Yuhua Jin

**Affiliations:** 1 School of Chemical Engineering, East China University of Science and Technology, Shanghai 200237, China; siya@starryink.com; 2 Shanghai StarryInk Biotechnology Co., Ltd., Shanghai 200080, China; yu@starryink.com; 3 School of Science and Engineering, The Chinese University of Hong Kong, Shenzhen 518172, China

**Keywords:** diarylethene derivatives, aggregation-induced emission effect, photophysical properties, photoresponsivity properties

## Abstract

Photochromic materials have attracted widespread attention due to their potential applications in optical information storage, optoelectronic devices, and fluorescence probes. As a typical photochromic system, diarylethene derivatives are considered one of the most promising photochromic materials due to their outstanding photostability and significant bistable properties. Based on an aggregation-induced emission (AIE) mechanism, this study employed a molecular structural engineering strategy to design and synthesize a series of diarylethene derivatives containing ethyl benzoate substituents. A systematic investigation of the structure–activity relationship between their photochromic behavior and AIE characteristics revealed a dual-state light response mechanism in the solid and solution states. This study demonstrates that the target compounds exhibited significant photochromic responses under UV–visible light irradiation, with enhanced emission in the solid state compared to the solution state, confirming the remarkable enhancement effect of AIE on aggregation. Structural characterization techniques such as nuclear magnetic resonance spectroscopy (NMR) and high-resolution mass spectrometry (H RMS) were employed to elucidate the correlation between molecular conformation and photophysical properties. Furthermore, these materials demonstrated potential for multi-level anti-counterfeiting, high-density optical storage, and bioimaging applications, providing experimental foundations for the development of novel multifunctional photochromic materials.

## 1. Introduction

Photochromism refers to a reversible transformation of molecular structures triggered by specific wavelengths of light, resulting in a shift in the absorption peaks, particularly in the visible region, and a corresponding color change [1]. This transformation often entails alterations in physical and chemical properties such as the luminescence intensity, redox potential, refractive index, dielectric constant, and magnetism [2,3,4,5]. Consequently, the use of photochromic materials has been explored for diverse applications, including optical switches, smart glasses, optical storage, molecular machines, logic gates, military uses, and anti-counterfeiting technologies [6,7,8]. Among photochromic materials, organic compounds such as spirooxazines, fulgides, spiropyrans, azobenzenes, schiff bases, and diarylethenes are particularly attractive due to their tunable structures and excellent photoresponsiveness [9,10,11,12]. Notably, diarylethene derivatives stand out for their thermal stability, high photoresponsivity, switching quantum yield, and fatigue resistance [13,14,15,16].

The utility of photochromic compounds continues to grow in applications beyond optical storage and logic circuits [17,18,19,20]. Emerging applications include photosensitive coatings, cosmetics, and 4D printing [21], as well as biological sensing. For example, Lin et al. (2025) engineered flexible organic crystalline fibers with a strong SHG (small molecule gelator) and mechanical flexibility via π-conjugated system design [22]. Shiraishi et al. developed spiropyran-based fluorescence sensors for cyanide detection in aqueous media [23,24]. In energy-efficient architecture, photochromic materials enable the production of smart windows that regulate light and heat, aided by electrochromic and thermochromic mechanisms [25,26]. Other developments include wearable photochromic fibers with a rapid UV response, durability, and potential utility in environmental and security applications, such as the WO_3_ nanorod-based composite developed by Wang et al. [27].

The reversible photochromic behavior of diarylethenes stems from a UV-induced cis–trans isomerization, which involves a small energy difference between isomers and rapid interconversion at room temperature [28]. Ongoing research has focused on enhancing diarylethene’s performance by introducing various substituents to improve its thermal stability, fatigue resistance, and fluorescence quantum yields [29,30,31,32,33,34]. While many traditional photochromic compounds suffer from aggregation-caused quenching (ACQ) due to π–π stacking or hydrogen bonding [35], aggregation-induced emission (AIE) offers a promising solution [36]. Tetraphenylethene derivatives, as representative AIEgens, are frequently employed due to their extended conjugation and electron-donating ability [37]. When co-integrated with diarylethene in hybrid molecules, these AIE-active units retain independent photophysical functions, allowing for the dual modulation of fluorescence through both molecular aggregation and light irradiation [38,39]. Such conjugated systems offer a powerful platform for developing advanced photoresponsive materials.

This study aimed to design and synthesize a series of photochromic diarylethene derivatives exhibiting AIE characteristics and thoroughly investigate their photochromic and AIE properties. Through molecular design, different substituents were introduced to modulate the photochromic behavior and luminescence properties of these molecules, elucidating the relationship between their molecular structure and performance. Additionally, the application potential of these materials in optical information storage, anti-counterfeiting, and fluorescence probes was explored, providing practical and experimental foundations for developing novel multifunctional photochromic materials.

## 2. Experimental Section

### 2.1. Materials

Potassium carbonate (K_2_CO_3_, AR, 99%), aluminum chloride (AlCl_3_, AR, 99%), zinc powder (AR, 99%), n-butyllithium (n-BuLi), titanium tetrachloride (TiCl_4_), toluene (AR, 98%), dichloromethane (DCM, AR, 99.5%), petroleum ether, tetrahydrofuran (THF, AR, 99%), and sodium chloride (NaCl, AR, 99.5%) were purchased from Shanghai Titan Scientific Co., Ltd., (Shanghai, China). Tetrakis(triphenylphosphine)palladium (Pd(PPh_3_)_4_, AR, 98%) and biphenyl dicarboxylic acid (AR, 98%) were obtained from Shanghai Bide Pharmatech Ltd., (Shanghai, China). N-Chlorosuccinimide (NCS, AR, 97%) and 2-methylthiophene (AR, 98%) were purchased from Shanghai Darui Fine Chemicals Co., Ltd., (Shanghai, China). Trimethyl borate (B(OMe)_3_, AR, 98%), benzoyl chloride (AR, 98%), ethyl 4-bromobenzoate (AR, 98%), and deionized water were obtained from Shanghai Macklin Biochemical Co., Ltd., (Shanghai, China). Unless otherwise specified, all reagents were used as received without further purification.

### 2.2. Instruments

^1^H NMR and ¹³C NMR spectra were recorded on a Bruker AV 400 nuclear magnetic resonance spectrometer (Bruker BioSpin GmbH, Rheinstetten, Germany) at room temperature, using deuterated chloroform (CDCl_3_) and deuterated dimethyl sulfoxide (DMSO-d_6_) as solvents, with tetramethylsilane (TMS, 0.03%) used as an internal standard. The fluorescence characteristics were analyzed using an SHG-200 handheld UV lamp (Shanghai Saiz Scientific Instrument Co., Ltd., Shanghai, China). Molecular structure confirmation was performed with a Shimadzu QP1000 high-resolution mass spectrometer (Shimadzu Corporation, Kyoto, Japan). UV-Vis absorption spectra were obtained using a Varian Cary 500 UV-Vis spectrophotometer (Agilent Technologies, Santa Clara, CA, USA). Fluorescence spectra were recorded on a Shimadzu RF-6000 fluorescence spectrophotometer (Shimadzu Corporation, Kyoto, Japan).

### 2.3. The Synthetic Route of the Target Compound, Diarylethene Ethyl Benzoate

**Figure 1:**
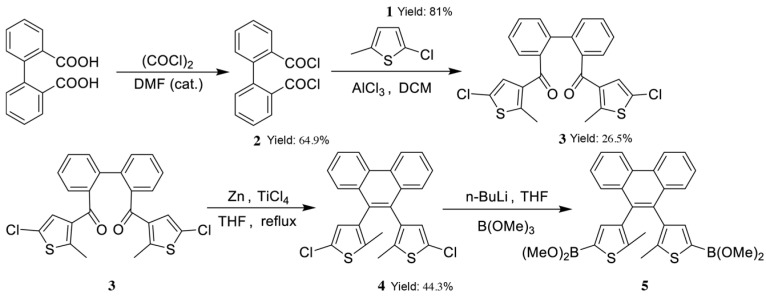
Synthesis route of diarylethene boronic ester.

The synthetic route of diarylethene boronic ester is illustrated in Figure 1. Starting with 2-methylthiophene as the raw material, chlorination was performed in THF using NCS under heating in the presence of glacial acetic acid to yield 2-chloro-5-methylthiophene (**1**) [40]. Biphenyl dicarboxylic acid was converted to biphenyl diacyl chloride (**2**) using benzoyl chloride in the presence of catalytic N,N-Dimethylformamide (DMF), which then underwent Friedel–Crafts acylation using anhydrous AlCl_3_ as a catalyst with 2-chloro-5-methylthiophene to yield a diketone intermediate (**3**) [41,42]. This diketone was subjected to McMurry coupling to form a phenanthrene-bridged diarylethene dichloride (**4**) [43,44]. The treatment of this compound with two equivalents of n-butyllithium (n-BuLi), followed by the addition of trimethyl borate (B(OMe)_3_), yielded a boronic ester intermediate (**5**) [45].

**Figure 2:**
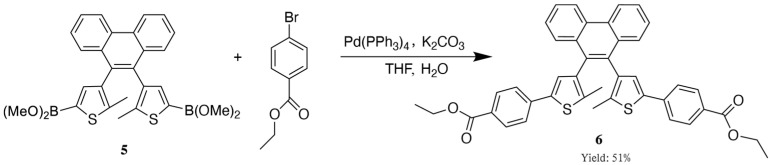
Synthesis route of diarylethene ethyl benzoate.

The synthetic route of diarylethene ethyl benzoate is shown in Figure 2. The boronic ester was used directly without purification in a Suzuki coupling reaction under basic conditions. In this step, tetrakis(triphenylphosphine)palladium (Pd(PPh_3_)_4_) was employed as the catalyst and potassium carbonate (K_2_CO_3_) as the base, and a mixed solvent system of water and THF was used under reflux conditions [46,47]. The coupling partner was ethyl 4-bromobenzoate, which introduced benzoate functional groups symmetrically on both sides of the phenanthrene bridge. This synthetic strategy yielded the desired diarylethene derivative featuring ethyl benzoate units with characteristic photoresponsive functionalities.

### 2.4. Synthesis of Diarylethene Ethyl Benzoate

The detailed synthetic procedures for intermediaries **1**–**4** are provided in the Supporting Information (SI). The boronic ester intermediate (**5**) was obtained by treating the dichloride precursor with n-butyllithium and trimethyl borate. It was directly subjected without purification to Suzuki coupling with ethyl 4-bromobenzoate under basic conditions, yielding the target diarylethene derivative containing photoresponsive ethyl benzoate groups.

**Figure 3:**
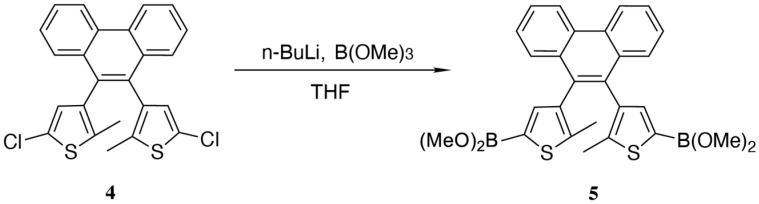
Synthesis of diarylethene boronic ester.

In a 25 mL flask, we pre-dried the derivative for a long time and then added a magnetic stir bar. We used a heat gun to bake it for 15 min while protecting it with an argon gas flow. We dissolved the previous product (0.5 mmol, 230 mg) in THF, added it to the reaction system under an ice–salt bath with a syringe, stirred the mixture for 5 min, and then added n-butyllithium (1.2 mmol, 0.48 mL) and stirred it for 30 min. We added trimethyl borate (1.5 mol, 0.117 g), removed the ice bath, and continued refluxing for 4 h. We did not perform any post-treatments and proceeded to the next experiment (Figure 3). ^1^H NMR (400 MHz, CDCl_3_) δ 8.81 (d, *J* = 8.3 Hz, 2H), 7.75–7.55 (m, 6H), 6.59 (s, 2H), 2.06 (s, 5H), 2.03 (s, 1H).

**Figure 4:**
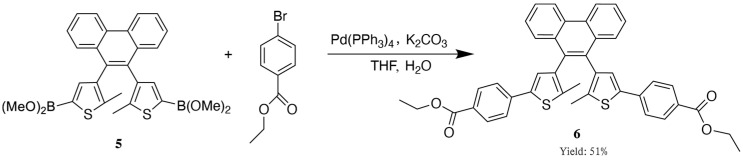
Synthesis of diarylethene ethyl benzoate.

In a 100 mL two-neck reaction flask, we added a magnetic stir bar, 4-bromobenzyl ester (2 mmol, 460 mg), and an aqueous potassium carbonate (10 mmol, 1.44 g) solution (5 mL). We added the solvent THF (15 mL) and tetrakis(triphenylphosphine)palladium (89 mg) and purged the system 2–3 times with an inert gas. We heated it to reflux at 60 °C and injected the product from the previous step (diarylethene boronic ester) using a syringe. We increased the temperature to 80 °C and refluxed the system overnight. The reaction mixture was poured into water to quench the reaction. The aqueous layer was extracted with ethyl acetate (3 × 20 mL), and the combined organic layers were dried over anhydrous Na_2_SO_4_. The solvent was removed under reduced pressure, and the crude product was purified by silica gel column chromatography (SiO_2_, ethyl acetate) to yield an off-white solid (168 mg, yield of 51%) (Figure 4). ^1^H NMR (400 MHz, CDCl_3_) δ 8.83 (d, *J* = 8.3 Hz, 2H), 7.97 (dd, *J* = 20.3, 8.5 Hz, 4H), 7.78–7.67 (m, 4H), 7.57 (dd, *J* = 9.6, 4.7 Hz, 2H), 7.50 (dd, *J* = 14.2, 8.5 Hz, 4H), 7.13 (d, *J* = 9.2 Hz, 2H), 4.37 (q, *J* = 7.1 Hz, 4H), 2.16 (d, *J* = 28.8 Hz, 6H), 1.40 (t, *J* = 7.1 Hz, 6H). ^13^C NMR (101 MHz, CDCl_3_) δ 166.32, 138.69, 138.61, 137.85, 137.58, 137.47, 137.14, 132.88, 131.45, 130.32, 130.21, 128.68, 128.20, 127.29, 127.11, 126.92, 126.73, 124.96, 124.84, 122.78, 60.95, 14.41, 14.32. H RMS (ESI): *m*/*z* [C_42_H_34_O_4_S_2_] calcd for [M + H]^+^: 689.1802; found 689.1796.

## 3. Results and Discussion

Diarylethene derivatives functionalized with specific groups possess both intrinsic photochromic properties and aggregation-induced emission (AIE) characteristics. However, the unmodified diarylethene core typically exhibits weak and irreversible photochromic responses. To overcome these limitations, functional groups were introduced at both termini to enhance the photoresponsiveness and optical reversibility of the molecular system. In this context, ethyl benzoate moieties were employed as functional substituents to improve the photochromic performance and promote molecular self-assembly, thereby enabling potential applications in supramolecular polymer-based materials.

Spectroscopic investigations revealed that the resulting compounds exhibited pronounced responses to ultraviolet and visible light irradiation. Their fluorescence emission and photochromic behavior were effectively modulated by external photostimulation. Furthermore, under UV irradiation, aggregation-induced emission phenomena were observed. Tetraarylethene-based structures incorporating ester linkages demonstrated reversible photochromism, indicating their suitability for use in further studies on light-responsive behavior under varying irradiation conditions.

All the synthesized compounds were found to undergo reversible photocyclization reactions upon alternating UV and visible light exposure. Accordingly, precursor structures were rationally designed with a variety of functional group substitutions to achieve the desired target molecules. The structural confirmation of the synthesized compounds was accomplished through ^1^H and ^13^C nuclear magnetic resonance (NMR) spectroscopy, while their optical properties were systematically examined by ultraviolet–visible (UV–Vis) absorption and fluorescence emission spectroscopy.

### 3.1. Photochromic Properties of Diarylethene Benzoate

**Figure 5:**
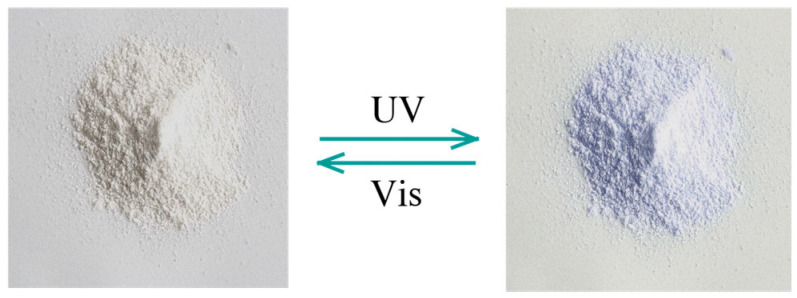
Color changes in diarylethene benzoate powder under UV/visible light.

The relationship between the structural changes and color transformation of diarylethene benzoate under UV light irradiation was investigated. As shown in Figure 5, the solid powder appeared milky white under ambient light. After 60 s of UV exposure, it turned pale blue-violet. Upon the removal of the UV light, the compound gradually returned to its original milky white state (Figure 5).

**Figure 6:**
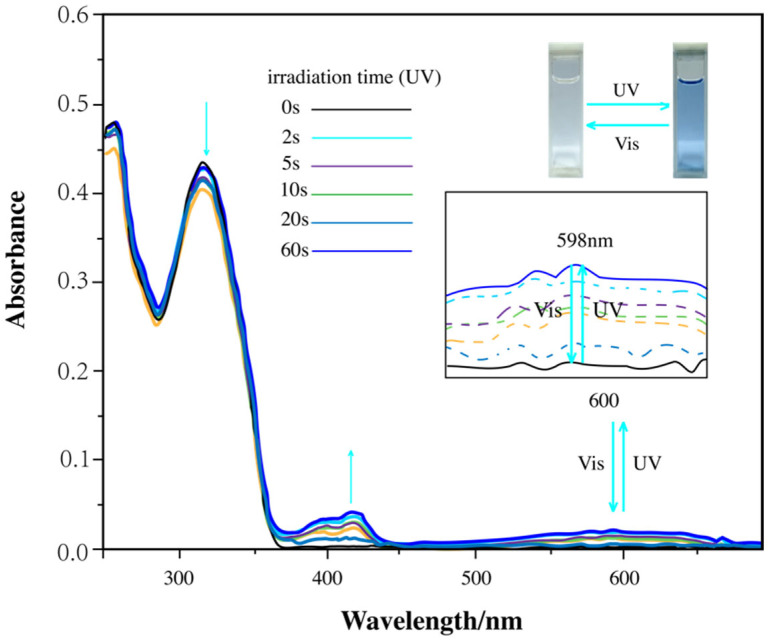
UV-Vis absorption spectrum showing changes in diarylethene benzoate in THF (2.0 × 10^−5^ M) under alternating UV/visible light irradiation.

To further verify this transformation, UV–visible absorption spectroscopy (Figure 6) was performed. Before UV irradiation, the compound in the THF solution exhibited an absorption peak at 335 nm, indicating an open-ring form. Upon 254 nm UV exposure, this peak decreased, and a new absorption band emerged at 598 nm. After 60 s of UV irradiation, the compound reached a photostationary state, characterized by an isosbestic point near 350 nm. This spectral change corresponded to the conversion of the colorless open-ring form into a blue closed-ring form. When the UV exposure was discontinued, the absorption spectrum returned to its initial state, and the solution regained its colorless appearance.

**Figure 7:**
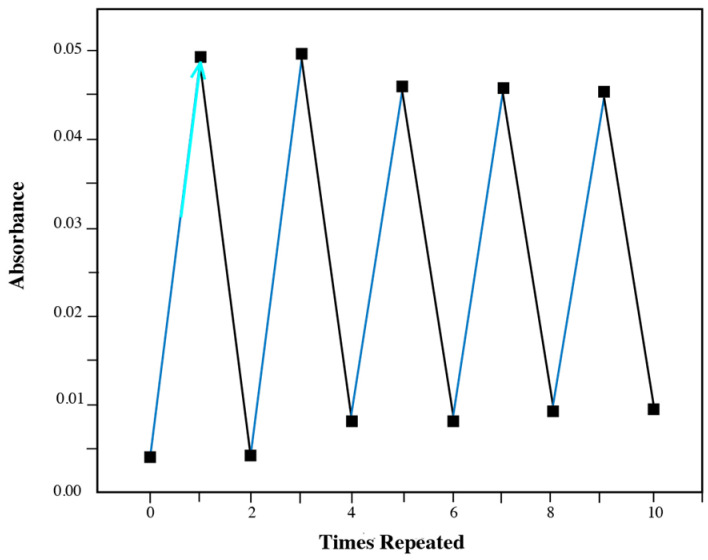
Absorbance changes in diarylethene benzoate solution under alternating UV/visible light.

The optical response was further confirmed through absorbance measurements (Figure 7). The absorbance of diarylethene benzoate changed significantly under alternating UV/visible light exposure, demonstrating good photoresponsiveness. Moreover, the photochromic effect remained robust even after 10 cycles of repeated switching, indicating high fatigue resistance.

### 3.2. Fluorescence Properties of Diarylethene Benzoate

**Figure 8:**
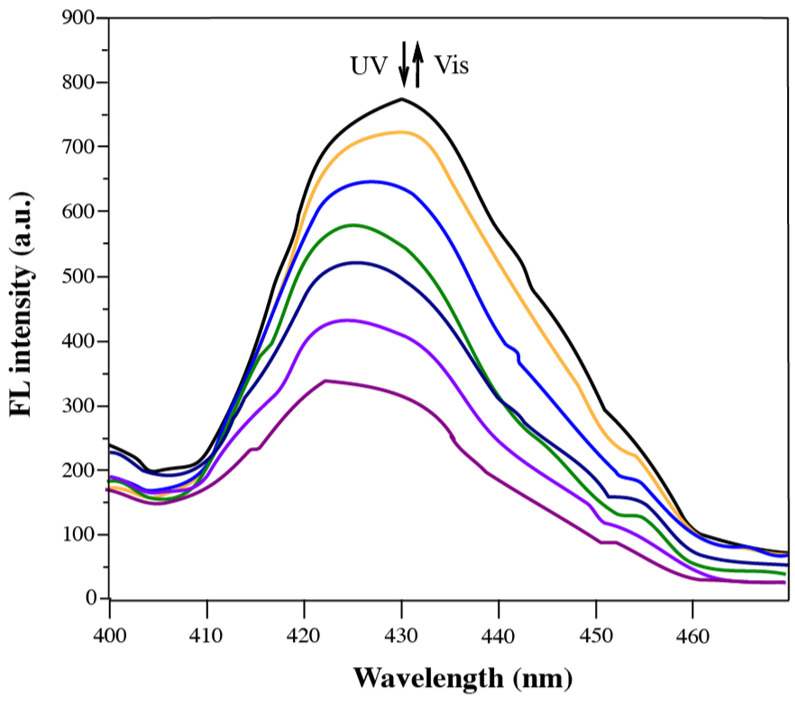
Changes in fluorescence emission intensity of solid diarylethene benzoate powder.

The fluorescence properties of solid-state diarylethene benzoate were investigated via fluorescence emission spectroscopy (Figure 8). As shown in Figure 8, the fluorescence intensity of the compound gradually decreased with prolonged UV irradiation, with the maximum emission observed at 420–435 nm. This reduction was attributed to the photoinduced structural transformation of the diarylethene moiety from its open-ring to closed-ring form, which increased the molecular planarity and enhanced π–π stacking interactions in the solid state [48]. Initially, the compound exhibited moderate solid-state fluorescence due to the restriction of intramolecular motion (RIM), a key mechanism of aggregation-induced emission (AIE) [49,50]. In this state, close molecular packing inhibited the torsional and vibrational freedoms of the aryl and thiophene groups, thereby minimizing non-radiative decay and promoting radiative transitions. However, as the UV irradiation proceeded and the molecular structure became more planar, excessive π–π stacking led to the formation of non-emissive aggregates. This aggregation-caused quenching (ACQ) effect was attributed to increased non-radiative relaxation pathways and exciton annihilation in the densely packed chromophoric system [51]. Additionally, a gradual red shift in the emission wavelength was observed, suggesting a reduction in the excited-state energy level due to stronger electronic coupling among the closely packed fluorophores. Such red-shifted emission behavior is commonly associated with excimer formation or extended conjugation upon photoisomerization [52]. These observations collectively indicate that the fluorescence behavior of diarylethene benzoate in the solid state is highly sensitive to structural and packing changes induced by photoirradiation.

**Figure 9:**
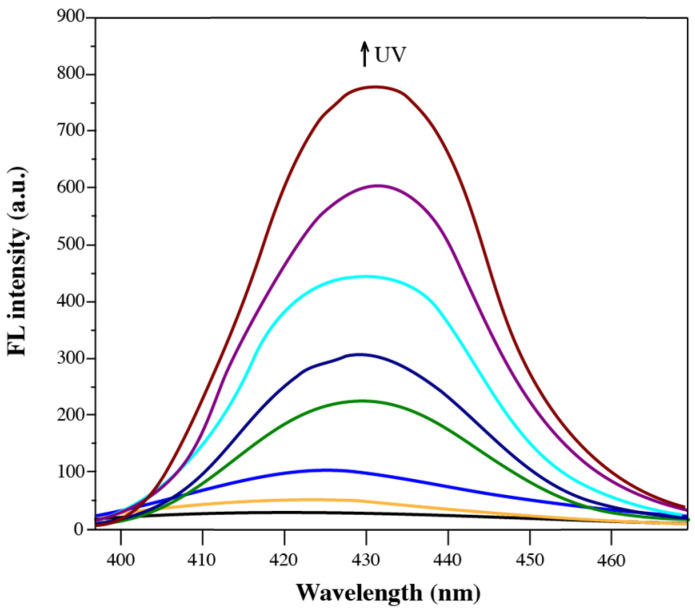
Changes in fluorescence emission intensity of diarylethene benzoate in THF solution (5.0 × 10^−5^ M).

In a pure THF solution, the compound initially exhibited no detectable fluorescence. However, upon exposure to UV irradiation, fluorescence emissions gradually emerged and intensified with an increasing irradiation time, reaching a photostationary state after approximately 60 s, with the maximum emission at 432 nm (Figure 9). This progressive enhancement in the fluorescence intensity was attributed to the ring-closing photoisomerization of the diarylethene core, which restricted the intramolecular rotation of the aromatic units. The restricted rotation facilitated molecular aggregation in solution, thereby triggering the aggregation-induced emission (AIE) effect. The time-dependent increase in emission confirmed the dynamic nature of the photoisomerization and aggregation processes, highlighting the compound’s responsiveness to UV stimuli in solution [53].

**Figure 10:**
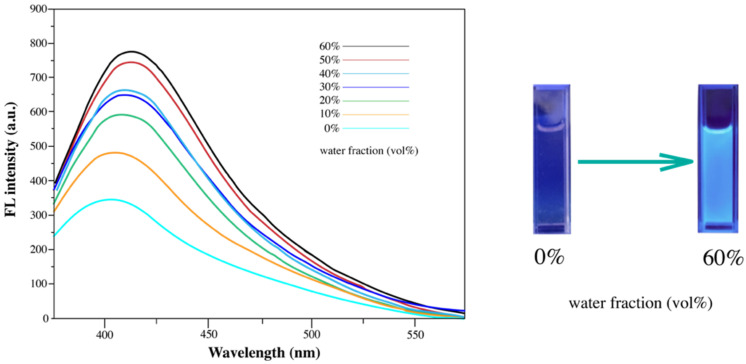
Changes in fluorescence emission intensity of diarylethene benzoate under alternating UV/visible light.

Fluorescence emission spectra were recorded in THF/water mixtures with varying water fractions to investigate the aggregation-induced emission (AIE) behavior of diarylethene benzoate (Figure 10). The compound was highly soluble in THF but poorly soluble in water, and in a pure THF solution (5.0 × 10^−5^ M), it exhibited almost no fluorescence. However, as the water content increased, the compound began to aggregate due to its limited solubility in aqueous media, resulting in a progressive enhancement in the fluorescence intensity. When the water fraction reached 60%, the emission reached a maximum at 432 nm, demonstrating a typical AIE effect [49,50]. The observed aggregation behavior can be attributed to both molecular and environmental factors. Structurally, the rigid and conjugated diarylethene core, along with the ethyl benzoate substituents, facilitated π–π stacking and enhanced the tendency toward aggregation in water-rich mixtures. In addition, the poor solubility in water induced hydrophobic interactions that drove self-assembly. These aggregation processes restricted the intramolecular rotations of peripheral aromatic units, thereby suppressing non-radiative decay and enabling strong fluorescence emission, a hallmark of AIE-active systems [54].

Although thermogravimetric and calorimetric data were not experimentally obtained in this study, we provide a structure-based thermal stability evaluation supported by precedents from the literature and a simulated thermoanalytical profile, as detailed in the Supporting Information [55,56,57].

### 3.3. Potential Application Discussion

Based on the structural features and photophysical responses of the synthesized diarylethene derivatives, their potential applications in advanced optical materials are worth highlighting. For example, recent work by Geng et al. has shown that structural color modulation using laser-based methods can achieve high-speed, full-color inkless printing, where the material’s color responsiveness plays a critical role in achieving precise, rewritable image encoding [58]. Similarly, Wessely et al. introduced a digital framework for optimizing photochromic materials in interactive 2.5D surface textures, highlighting the importance of a rapid, reversible optical contrast in rewritable printing applications [59]. In this context, the relatively high absorption band and photoresponsivity of our system, combined with its cyan-blue emission characteristics, could contribute to color tuning in printable substrates. Compared with conventional cyclopentene-type diarylethenes, the current system may offer advantages in terms of synthetic accessibility and cost-effectiveness, while covering a wider color gamut.

Moreover, the integration of fluorescence modulation into logic-based or security-relevant devices is becoming increasingly relevant. Recent studies have demonstrated the use of multi-level anti-counterfeiting platforms using photoresponsive quantum dot–porphyrin systems [60] and AIE polymers capable of simultaneously supporting data storage and dynamic fluorescence coding [61]. Notably, Zhang et al. have shown that AIE-active luminogens can be engineered to achieve petabit-level optical storage, leveraging their reversible fluorescence behavior and high signal contrast under optical excitation [62]. The bistable optical switching and high fatigue resistance of our material could allow for similar incorporation into future anti-counterfeiting or optical encryption platforms.

Finally, the potential of AIE-active materials in biological imaging has been well documented. These observations suggest that this class of compounds could be further adapted for bioimaging or sensing purposes, as has been demonstrated for AIE-active nanomaterials with high solid-state fluorescence and photoswitchability [63,64], suggesting that this class of compounds could be further adapted for imaging or sensing purposes in the future.

## 4. Conclusions

This study successfully integrated an ethyl benzoate unit into a phenanthrene-bridged dithienylethene system, resulting in the synthesis of diarylethene benzoate with dual photochromic and aggregation-induced emission (AIE) properties. UV–visible absorption and fluorescence emission spectroscopy demonstrated significant reversible photochromic responses under UV–visible light stimulation. Solid-state samples exhibited fluorescence quenching upon UV irradiation, while THF/water mixed solvent systems displayed strong AIE effects. Furthermore, fatigue resistance testing verified the excellent reversible photochromic performance and cycling stability of this phenanthrene-bridged diarylethene system. The experimental results confirmed that the designed molecular system successfully integrated photochromic functions with AIE characteristics. Future studies could explore the self-assembly behavior of this molecule and its potential applications in smart responsive materials.

## Data Availability

The original contributions presented in this study are included in the article/Supplementary Materials. Further inquiries can be directed to the corresponding author(s).

## Supplementary Materials

The following supporting information can be downloaded at: https://www.mdpi.com/article/10.3390/ma18112520/s1, Figure S1: Synthesis of 2-3 2-chloro-5-methylthiophene; Figure S2: Synthesis of biphenyl-2,3-dicarboxylic acid chloride; Figure S3: Synthesis of 2,2′-bis(5-chloro-2-methylthiophene-3-carbonyl)-biphenyl; Figure S4: Synthesis of 9,10-bis(5-chloro-2-methylthiophene)-3-phenanthrene; Figure S5: ^1^H NMR spectrum of diarylethene ethyl benzoate; Figure S6: ^13^C NMR spectrum of diarylethene ethyl benzoate; Figure S7: H RMS spectrum of diarylethene ethyl benzoate; Figure S8: Simulated TGA and DSC Curves of diarylethene ethyl benzoate.
