# Supplementary material for: Synthesis and Photochromic Properties of Diarylethene Derivatives with Aggregation-Induced Emission (AIE) Behavior
Source: Materials (Basel). 2025 May 27;18(11):2520. doi: 10.3390/ma18112520 (PMC12156935; doi:10.3390/ma18112520)
Supplement: Supplementary file 1 [file materials-18-02520-s001.zip › materials-3628890-supplementary.pdf]

Synthesis and Photochromic Properties of Diarylethene Derivatives with Aggregation-Induced Emission (AIE) Behavior

Jiaxin Guo [1], Haoyuan Yu [2] and Yuhua Jin [3,*]

[1] School of Chemical Engineering, East China University of Science and Technology, Shanghai 200237, China; siya@starryink.com
[2] Shanghai StarryInk Biotechnology Co., Ltd., Shanghai 200080, China; yu@starryink.com
[3] School of Science and Engineering, The Chinese University of Hong Kong, Shenzhen, 518172, China
* Correspondence: yuhuajin@cuhk.edu.cn

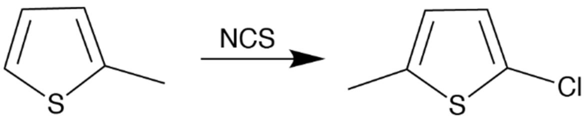

1

Figure S1. Synthesis of 2-3 2-chloro-5-methylthiophene.

Weigh 60.0 g (0.61 mol) of 2-methylthiophene and add it all at once to a 500 mL three-necked flask. Add a magnetic stir bar, 300 mL of solvent (acetic acid and 120 mL of THF), and protect the reaction system with an argon gas flow. Add N-Chlorosuccinimide (0.72 mol, 95.76 g) and stir at room temperature for 30 minutes. Then, carry out a reflux reaction for 4 hours. Afterward, add an appropriate amount of ice water to quench the reaction. Extract the aqueous phase three times with dichloromethane, and wash with saturated brine. Combine the organic phases and dry them using anhydrous sodium sulfate. Remove the dichloromethane under rotary evaporation and then distill under reduced pressure to obtain a colorless liquid（70.05g, yield 81%）[40]. ^{1}H NMR (400 MHz, $CDCl_3$) δ 2.31 (s, 3H), 6.43 – 6.41 (m, 1H), 6.59 (d, J = 4.0 Hz, 1H).

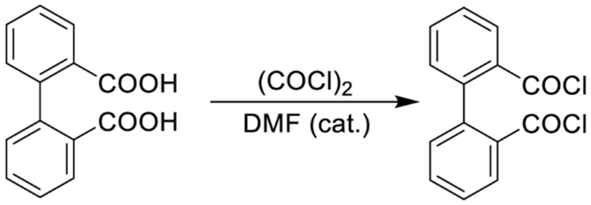

2

Figure S2. Synthesis of biphenyl-2,3-dicarboxylic acid chloride.

In a 100 mL dry two-necked flask, dissolve biphenyl-2,3-dicarboxylic acid (18 mmol, 4.36 g) in DCM (50 mL), and add oxalyl chloride (22 mmol, 3.17 g). Under an ice-salt bath, add a catalytic amount of DMF(0.5mL) and stir at room temperature for 4 hours.

Then, remove the solvent under rotary evaporation to yield a yellow-green solution of the product (1.56g, yield 64.9%) [41]. ¹H NMR（$CDCl_3$, 400 MHz）δ 8.79 (d, J = 8.1 Hz, 2H) δ 7.55–7.73 (m, 6H) δ 6.56–6.57 (s, 2H).

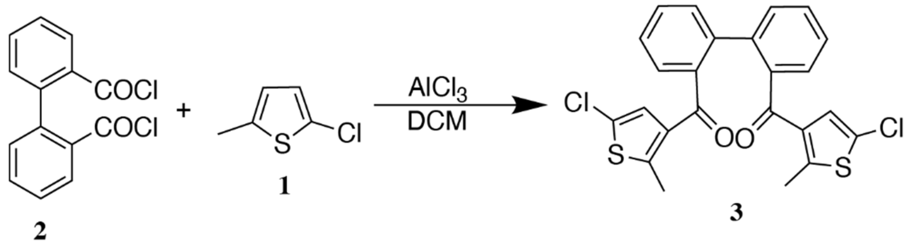

Figure S3. Synthesis of 2,2'-bis(5-chloro-2-methylthiophene-3-carbonyl)-biphenyl.

In a 50 mL dry reaction flask, add a magnetic stir bar, and then add biphenyl-2-carbonyl chloride to the DCM solution (50mL) of 2-chloro-5-methylthiophene (2.39g, 18mmol). After maintaining the mixture under an ice-salt bath for a while, add aluminum chloride (3.0g, 22.5mmol) in batches. Stir for 30 minutes under an ice bath, then remove the ice-salt bath and continue the reaction at room temperature for 16 hours [42]. The reaction mixture was quenched with ice-water to remove excess $AlCl_3$, followed by extraction with ethyl acetate (3 × 20 mL). The combined organic layers were washed with saturated brine, dried over anhydrous Na_2SO_4, and concentrated under reduced pressure. The crude product was purified by silica gel column chromatography (SiO_2, ethyl acetate) to afford a white solid. (1.11g, yield 26.5%)¹H NMR (400 MHz, $CDCl_3$) δ 7.57 – 7.48 (m, 2H), 7.43 – 7.35 (m, 6H), 6.74 (s, 2H), 2.50 (s, 6H).

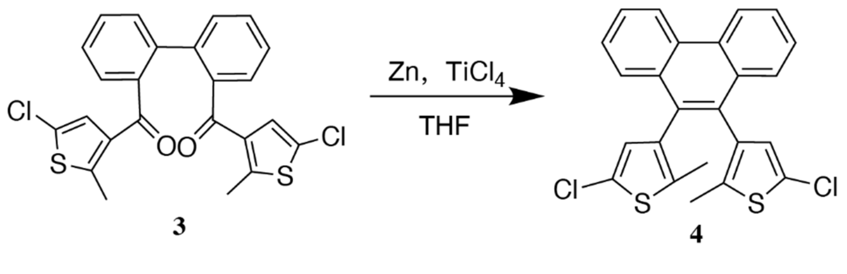

Figure S4. Synthesis of 9,10-bis(5-chloro-2-methylthiophene)-3-phenanthrene.

In a 100 mL flask, add zinc powder (4.42 mmol, 0.2873 g) and a magnetic stir bar, and use an argon gas flow to protect and vent. Add THF as the solvent. Under an ice-salt bath, slowly add titanium tetrachloride (3.39 mmol, 0.37 mL), maintain the reaction for 15 minutes, then reflux for 1 hour. Add the intermediate product and continue refluxing for 4 hours [43-44]. The reaction mixture was poured into an aqueous solution of K_2CO_3 to quench the reaction, followed by extraction with dichloromethane (3 × 20 mL). The combined organic layers were dried over anhydrous Na_2SO_4 and concentrated under reduced pressure. The resulting crude product was purified by silica gel column chromatography (SiO_2, ethyl acetate 25:1) to afford the target solid. (230mg, yield

44.3%).^{1}H NMR (CDCl3, 300 MHz) δ 2.00, 2.04 (2s, 6H), 6.56, 6.57 (2s, 2H), 7.55 – 7.73 (m, 6H), 8.79(d, J = 8.1Hz, 2H),

NMR and Mass Spectrometry Analysis of Diarylethene Benzoate

To confirm the molecular structure of the synthesized compound, 1H NMR, 13C NMR, and high-resolution mass spectrometry (H RMS) analyses were performed. The corresponding spectral data are presented.

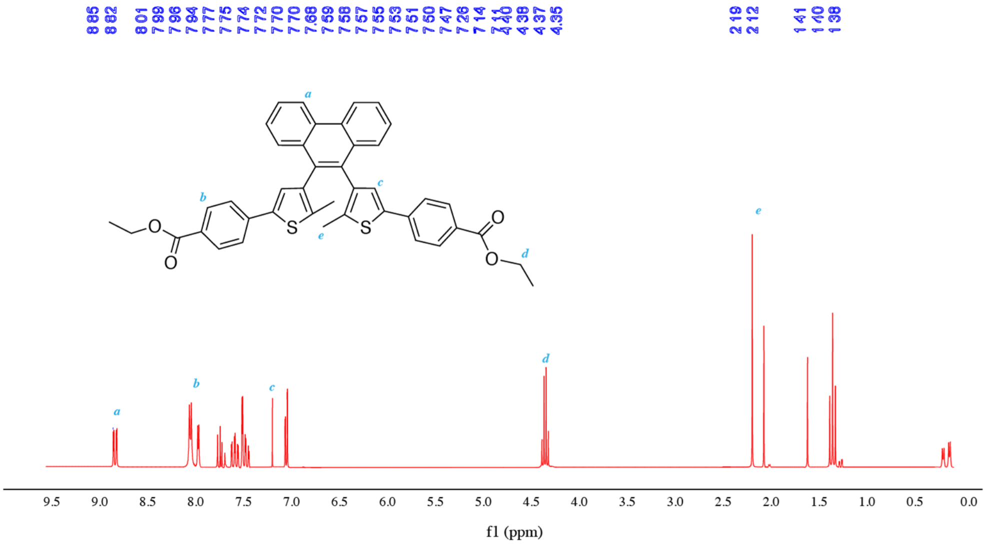

Figure S5. ^{1}H NMR spectrum of diarylethene ethyl benzoate.

In the ^{1}H NMR spectrum, using deuterated chloroform as the solvent, both parallel and anti-parallel conformations of the target compound are observed. The signals of these two isomers almost overlap in the downfield region but exhibit significant differentiation in the up-field region, resulting in a complex spectral pattern. The doublet at 8.83 ppm corresponds to the protons on the phenanthrene bridge. The aromatic protons from the benzoate units attached to the diarylethene moiety appear as multiples at 7.94-8.01 ppm and 7.47-7.53 ppm. The six protons at 7.55-7.77 ppm are attributed to the remaining phenanthrene bridge protons. The singlet at 7.11-7.14 ppm corresponds to the thiophene shoulder proton. The quartet at 4.35-4.40 ppm represents the methylene protons of the ester group, while the singlets at 2.19 ppm and 2.12 ppm (six protons) correspond to the methyl groups on the thiophene rings. Finally, the triplet at 1.41-1.38 ppm (six protons) corresponds to the methyl groups at the benzoate termini.

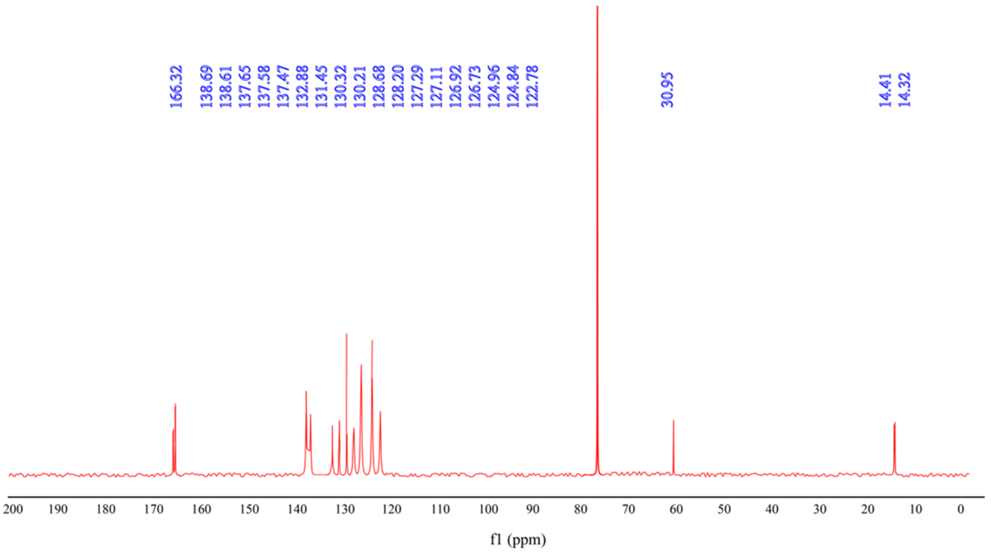

Figure S6. ^{13}C NMR spectrum of diarylethene ethyl benzoate.

From the ^{13}C NMR spectrum, the characteristic peaks at 14.32 ppm and 14.41 ppm were observed, which correspond to the methyl groups, one from the thiophene ring and the other from the benzoate terminus. The singlet at 60.95 ppm represents the methylene carbon in the benzoate group. The characteristic peak at 166.32 ppm corresponds to the carbonyl carbon of the benzoate moiety. Multiple peaks between 122.78 ppm and 138.69 ppm are characteristic of the aromatic rings in the diarylethene structure. The NMR data are consistent with the expected molecular structure, confirming the successful synthesis of the target compound.

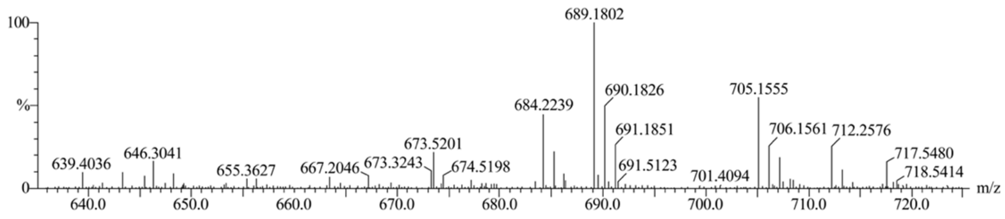

Figure S7. H RMS spectrum of diarylethene ethyl benzoate.

The high-resolution mass spectrum (H RMS) exhibits a molecular ion peak at $[M+H]^{+}$ = 689.1802, which is in excellent agreement with the calculated mass of 689.1796, confirming the molecular composition and accuracy of the synthesized compound . The M+2 peak at m/z 691.1851 is attributed to the natural isotope distribution of sulfur (^{34}S), consistent with the presence of two sulfur atoms in the molecular formula and theoretical predictions (approx. 8.4% relative intensity). The peak at m/z 655.3627 may correspond to a trace deborylated by-product ($C_{38}H_{28}O_4S_2$), possibly resulting from partial degradation of the boronic ester intermediate during the Suzuki coupling.

Thermal Stability Estimation Based on Structural Analogues and Simulated Thermoanalytical Behavior

Although thermogravimetric (TGA) and differential scanning calorimetry (DSC) measurements were not conducted in this study due to time and equipment constraints, we evaluated the thermal behavior of the synthesized diarylethene benzoate via structural analysis and literature comparison. He (2014) reported a series of phenanthrene- and perylene-bridged diarylethenes with ester and ketone substituents that remained thermally stable above 300 °C. Several of these compounds also formed single crystals, suggesting high molecular rigidity and solid-state robustness. Notably, benzoate-functionalized derivatives showed consistent photochromic switching under repeated UV/Vis cycles, indicating good thermal and optical stability [56].

Our compound shares these key features, including a rigid phenanthrene bridge, two thiophene rings, and ethyl benzoate groups. This extended conjugation not only enhances AIE and photochromism but also contributes to thermal resilience. Benzoate esters are known to remain stable up to 250–300 °C under inert conditions, and related AIE systems have exhibited comparable thermal robustness [57].

To estimate the thermal response, we generated simulated TGA and DSC curves based on structure-property correlations. As shown in the figure below, the TGA simulation predicts minimal mass loss below 250 °C, gradual decomposition up to 450 °C, and rapid degradation beyond that. The DSC profile indicates a broad endothermic transition around 180–200 °C—possibly a glass transition—and an exothermic decomposition peak near 470 °C [58].

These results suggest that the diarylethene benzoate derivative exhibits promising thermal stability for potential integration into photoresponsive devices. Experimental TGA/DSC characterization is planned to confirm these trends and elucidate the decomposition mechanism.

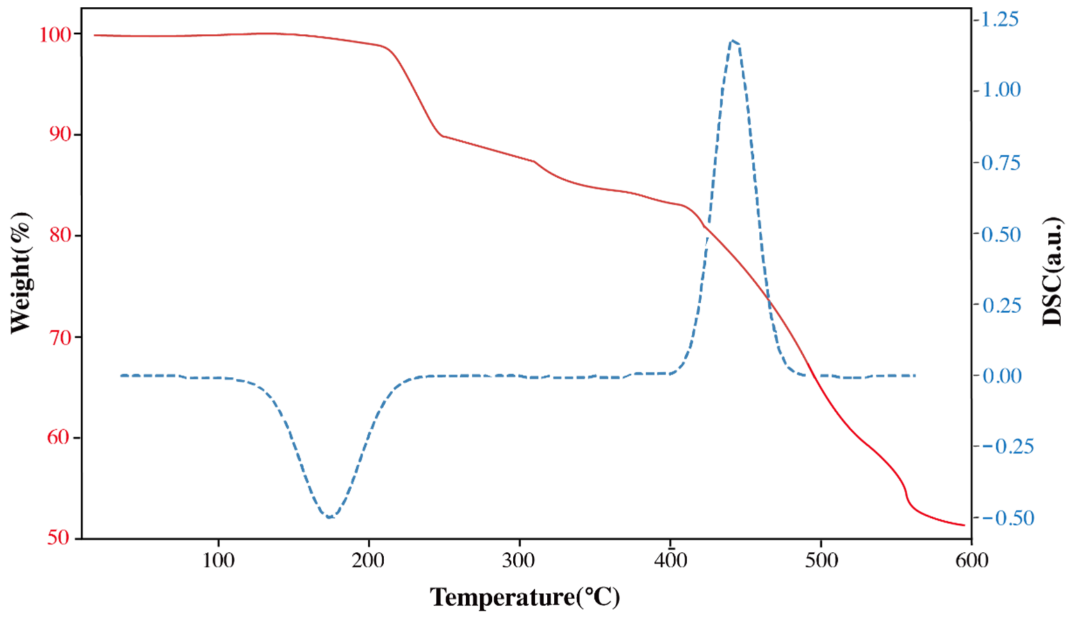

Figure S8. Simulated TGA and DSC Curves of diarylethene ethyl benzoate.

References

40. Luo, Q.; Fan, Q.; Huang, W. Synthesis overview of diarylethene-based photochromic materials. *J. Org. Chem.* **2007**, *02*, 175–187.
41. Nishimura, R.; Nagakawa, Y.; Morimoto, M. Multicolor photochromism of two-component diarylethene crystals containing oxidized and unoxidized benzothiophene groups. *Crystals* **2022**, *12*, 1730. https://doi.org/10.3390/cryst12121730.
42. Zhai, S.; Zhu, X.; Zeng, H. Synthesis and properties of 1,3,4-oxadiazole-linked diarylethene compounds. *J. Chin. Univ. Chem.* **2011**, *32*, 2316–2320.
43. Li, H.; Lu, D.; Qian, B.-Y.; Lin, J.; Zhang, H.-J. Direct synthesis of K-region functionalized polycyclic aromatic hydrocarbons via twofold intramolecular C–H/C–H arylation. *Org. Lett.* **2024**, *26*, 11140–11144. https://doi.org/10.1021/acs.orglett.4c04156.
44. Cheng, H.; Zhang, G.; Cheng, R.; Li, Z. F.; Luo, Q. F. Synthesis and properties of multi-unit diarylethene photochromic compounds. *Chin. J. Appl. Chem.* 2011; 28. （Suppl）,1–5.
45. Spangenberg, A.; Métivier, R.; Gonzalez, J.; Nakatani, K.; Yu, P.; Giraud, M.; Léaustic, A.; Guillot, R.; Uwada, T.; Asahi, T. Multiscale approach of photochromism: Synthesis and photochromic properties of a diarylethene in solution, in nanoparticles, and in bulk crystals. *Adv. Mater.* **2009**, *21*, 309–313. https://doi.org/10.1002/adma.200801578.
55. He, J.J. Photophysical and Chemical Properties of Phenanthrene- and Perylene-Based Diarylethene Photochromic compounds. Ph.D. Thesis, South China University of Technology, Guangzhou, China, 2014.
56. Hu, Z.; Zhang, H. K.; Chen, Y.; Wang, Q. S.; Elsegood, M. R. J.; Teat, S. J.; Feng, X.; Islam, M. M.; Wu, F. G.; & Tang, B. Z. Tetraphenylethylene-based color-tunable AIE-ESIPT chromophores. *Dyes Pigm.* **2020**, *175*, 108175. https://doi.org/10.1016/j.dyepig.2019.108175.
57. Patel, D.G.; Walton, I.M.; Cox, J.M.; Gleason, C.J.; Butzer, D.R.; Benedict, J.B. Photoresponsive porous materials: The design and synthesis of photochromic diarylethene-based linkers and a metal–organic framework. *Chem. Commun.* **2014**, *50*, 2653–2656. https://doi.org/10.1039/C3CC49666J.
